# Postpartum Uterine Involution and Embryonic Development Pattern in Chinese Holstein Dairy Cows

**DOI:** 10.3389/fvets.2020.604729

**Published:** 2021-01-22

**Authors:** Yuxin Lin, Hongzhen Yang, Muhammad Jamil Ahmad, Yuze Yang, Wucai Yang, Hasan Riaz, Adili Abulaiti, Shujun Zhang, Liguo Yang, Guohua Hua

**Affiliations:** ^1^Key Lab of Agricultural Animal Genetics, Breeding and Reproduction of Ministry of Education, College of Animal Science and Technology, Huazhong Agriculture University, Wuhan, China; ^2^Beijing General Station of Animal Husbandry, Beijing, China; ^3^College of Animal Science, Northwest A&F University, Yangling, China; ^4^Department of Biosciences, COMSATS University Islamabad, Sahiwal, Pakistan

**Keywords:** Chinese Holstein cow, uterine involution, parity, age, embryo development

## Abstract

Understanding the postpartum uterine involution pattern and embryonic development could facilitate bovine reproduction management, improve reproductive efficiency, and diagnosis of the reproductive disorder, which would contribute to the success of the dairy business. This study aimed to investigate postpartum uterine involution and embryonic developmental patterns or postconceptional marks of embryonic fetal development in Chinese Holstein dairy cows using B-mode ultrasonography. The results revealed a significant decline in the involution period with an increase of parity and age. The uterine involution period was shorter in multiparous cows when compared with cows with lower parities. Consistently, cows over 4 years old recovered faster than younger cows (2 or 3 years). Besides, the elder cows (over 4 years) had a relatively larger size of resumed cervix uteri and horns. Postpartum uterine involution pattern analysis revealed that the reproductive tract recovered very fast during the first 16 days postpartum for all the parity. Results of postconceptional marks of embryo development revealed a slow increase in diameter of the gravid uterine horn and crown-rump length (CRL) before day 60. In contrast, this increase was dramatic and rapid after the 60th day. We also established two models to estimate gestational age based on gravid uterine horn diameter or CRL. A formula was established to determine the gravid uterine horn size during postconceptional on day 30th–day 90th (*r* = 0.8714, *P* < 0.01). In addition, a significant positive correlation between CRL and gestational age (*r* = 0.98151, *P* < 0.01) was built. In conclusion, these results illustrated that parity and calving age had significant effects on uterine involution in Chinese Holstein cows. Crown-rump length and gravid uterine horn diameter are both efficient for evaluating the embryo growth. These current findings broaden the understanding of basic reproductive pattern in Chinese Holstein cows and could benefit bovine reproductive management primarily in postpartum and early pregnant cows to reduce the calving interval and avoid periparturient metabolic diseases.

## Introduction

The reproductive performance of dairy cows determines the profitability and success of the dairy business with the ideal goal of achieving one calf per cow per year (1). Postpartum uterine involution is very critical to the recurrence of the reproductive cycle and next pregnancy (2, 3). Whether uterine involution has been successful or abnormal is a precondition for artificial insemination in bovine breeding (4). Uterine involution is a physiological process by which the uterus turns to its pre-pregnancy dimensions with endometrial regeneration, reduced uterine blood flow and endometrial vascularity, and reduced muscle mass (5, 6). Several physiological factors affect the uterine involution in dairy cows such as breeds, nutritional conditions, body condition score (BCS) at calving, and postpartum diseases (7–10). There are, however, contradictory reports of the parity-uterine involution relationship. Parity effects on restoring ovarian function suggest that primitive cows take longer than multiparous cows (11). In contrast, Miettinen (12) reported that the parity of Finnish dairy cows has no significant effect on the duration for complete uterine or cervical involution. The interval from calving to the first ovulation becomes progressively longer as the number of parity increased in Friesian cows (13). Therefore, further investigations of uterus, cervix, and horn are necessary to confirm whether parity and calving age influence uterine involution.

Reproductive performance in dairy cows continues to decline as characterized by low fertilization rates and reduced embryonic survival (14). Pregnancy loss during early embryo and fetal development is one of the leading causes of reproductive failure (15, 16). Pregnancy loss in cattle mainly occurs in the first 40 days of gestation, and fetal loss after 42 days of gestation is less than 10% (17). Therefore, the detection of pregnancy is of great economic importance for the cattle industry (18). Ultrasound has been widely used for cattle reproduction and has provided the practitioners with a way to gather more information than via rectal palpation (19–21). Ultrasonography is also a useful tool for the early pregnancy diagnoses and embryo development study. The Crown-rump length (CRL) initially used to estimate gestational age prediction (22), is an indicator of good reproductive management of dairy cows, including predicting parturition date, designing a better mating plan, and collection of reproductive data of new cattle to increase the benefit-cost ratio (18, 23, 24). CRL's equations have proved to estimate fetal age accurately (18, 25, 26). However, the relationship between the postconceptional increase in uterine horn diameter and early fetal development is still not clear. Therefore, further investigations of uterus horn and early embryo development after gestation are necessary to analyze the reasons for low embryonic survival rates and making appropriate reproductive management plans.

Since 1992, Chinese Holstein (Chinese Black and White cows produced by mating the Holstein to native Chinese) are recognized by China's Ministry of Agriculture (27). Chinese Holstein is the major dairy cattle breed in China, providing nearly 90% of Chinese milk production (27), with an annual milk potential of 4,500–10,000 kg milk with 3.5% fat (27). While many studies have been conducted worldwide on reproductive performance and longevity in dairy cows, to date few have focused on Chinese cows, particularly from southern China.

This study investigated the effects of parity and age on the involution period by monitoring the resumption pattern of the reproductive tract to standard non-pregnant size in postpartum Holstein dairy cows. Furthermore, we established two models to evaluate the gestation age and postconceptional marks of embryonic development from day 30th−90th in Holstein dairy cows.

## Materials and Methods

### Animals and Management

All experimental techniques were performed following the guidelines of the Committee of Animal Research Institute, Huazhong Agricultural University, China. The Ethical Committee of the Hubei Research Center of Experimental Animals approved this study [Approval ID: SCXK (Hubei) 20080005].

In the present study, Holstein dairy cows (*n* = 109) with body condition score (BCS) of 3.0–3.5 were divided into 3 groups named: primiparous (*n* = 28), biparous (*n* = 20), and multiparous (*n* = 61, 3rd (*n* = 24), 4th (*n* = 17), and 5th−9th parity (*n* = 20). BCS of a dairy cow is a visual assessment of the proportion of body fat and muscles covering the bones of a cow, recognized by animal scientists and producers as being an important factor in dairy cow's managements. Missing data related to milk yield and negative energy balance is the only limitation that was handled by enrolling the cows of BCS (3.0–3.5). All the experimental animals were free from clinical uterine infection, periparturient diseases, calving difficulty, and other general reproductive problems. The experimental animals were raised on a local dairy farm in the south of China. The cows received an adequate amount of total mixed ration per day to meet the energy needs for milk yield and maintain BCS. The location of the farm was between 30° 44′ N and 114° 23′ E, at an average altitude of 50 meters above sea level. The average annual rainfall in the experimental area was 1,290 mm, with an average temperature of about 17.2°C, with minimums of −5°C and maximums of 36°C.

### Ultrasonographic Observation

**Figure 1:**
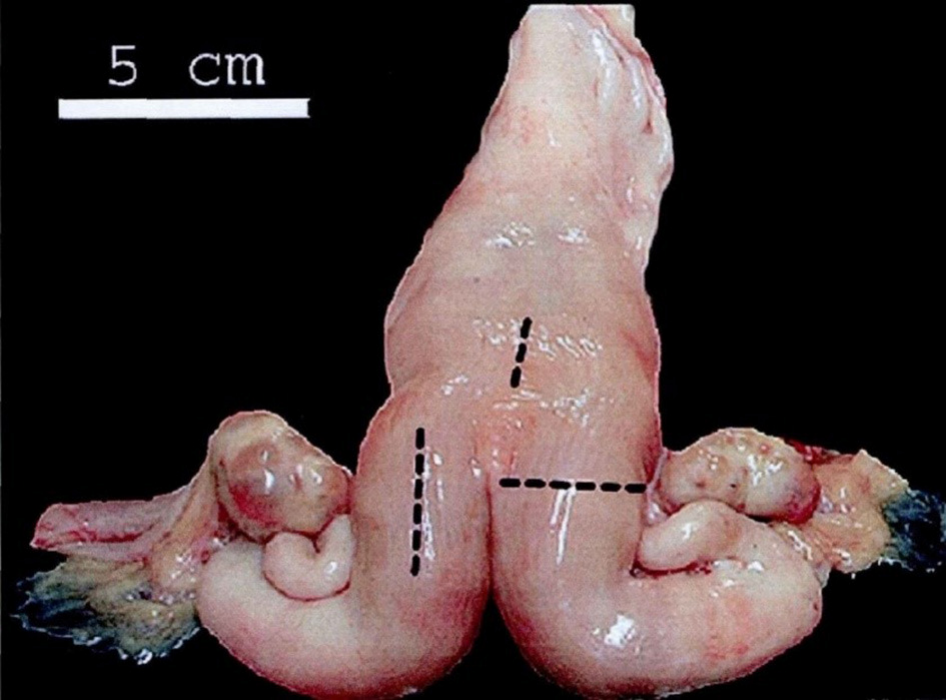
Anatomical model of the bovine uterus. Black lines mark the locations where the B-mode images of the uterus were taken. One long-sectional image of each uterine horn and one long-sectional image of the cranial end of the cervix were taken using a B-mode veterinary ultrasound scanner in our study. The figure is cited from Schmauder et al. (30).

The rectal ultrasonography of the uterus was undertaken with a B-mode veterinary ultrasound scanner (LV2-2/6.5 MHZ, linear array transducer, Shenzhen, China). Photographs of the uterus were transferred to a computer workstation for analysis to assess the extent of uterine involution at different postpartum days according to the method established in our laboratory (2, 28, 29). Before the examination, the dung was gently removed from the rectum, and the probe was inserted and placed on the uterus. The same operator performed all ultrasound examinations to avoid system error. Long-sectional images were obtained by placing the probe in a longitudinal direction. Then, the images were frozen and inspected to be measured (Figure 1) described previously (30).

Different measurements of reproductive tract and ultrasonography were started on the 7th day after calving and continued every 3rd day till the completion of uterine involution. The uterine horns were considered symmetrical when their diameters were within 1 cm of one another, and no further changes in diameter could be differentiated during two successive examinations (31).

**Figure 2:**
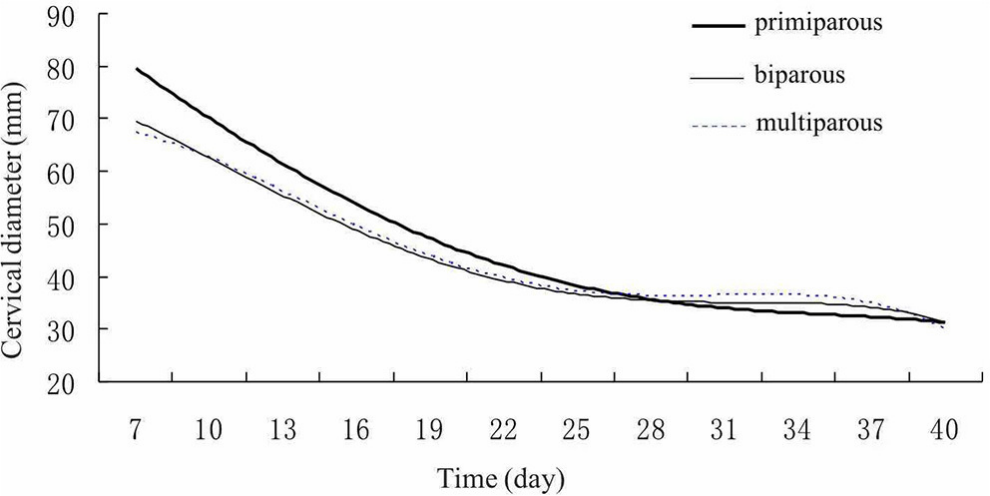
Cervix Uteri involution pattern of Holstein cows. Cervix Uteri diameters gradually declined in different parities of cows after calving.

**Figure 3:**
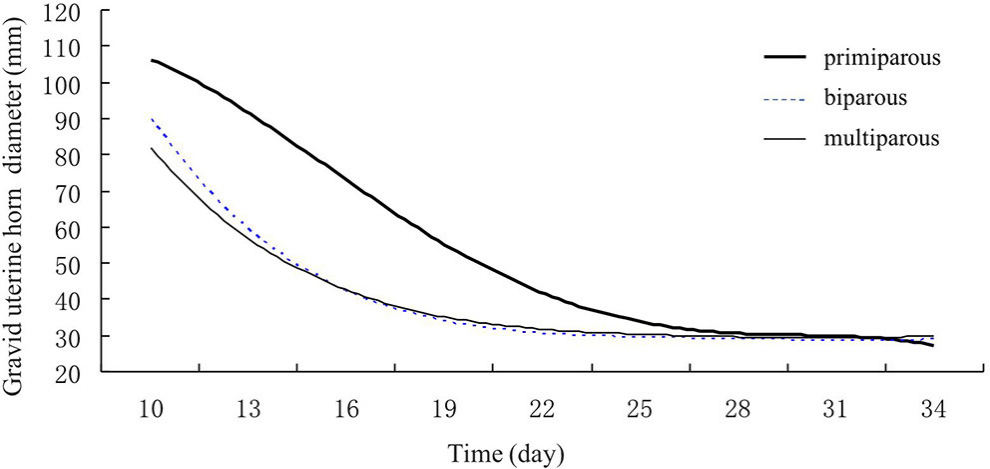
Gravid uterine horn involution of primiparous, biparous, and multiparous Holstein cows. Gravid uterine horn diameters of biparous and multiparous cows decreased faster than those of primiparous cows.

In addition, the abdominal ultrasound scans (LV2-2/5.0 MHz, transducer, Shenzhen, China) were used to measure Crown-rump length (CRL) (the distance from the top of the head to the end of the tail of the fetus) proposed by Robinson (22). For the postconceptional change in diameter of uterine horns and CRL ultrasonography, observations were taken from day 30th to day 90th post insemination with an interval of 30 days. Ultrasound scans revealed the reproductive tract structures in transverse and longitudinal sections to evaluate the echogenicity and echotexture of these tissues. Finally, regression curves were analyzed to show the postpartum involution pattern of the reproductive tract in Holstein cows (Figures 2, 3).

### Statistical Analysis

Experimental data were presented as mean ± S.EM. The one-way analysis of variance (ANOVA) was used to determine the statistically significant difference among the experimental groups by the SAS program (version 9.2, SAS Institute Inc., Gary, NC, USA) using *post-hoc* Tukey's multiple comparisons tests (*P* < 0.05). A probability of *P* < 0.05 was considered statistically significant.

For regression analysis, fetal parameters (CRL) and gravid uterine horn diameter were considered as dependent variables and gestational age as independent variables. The equation of the linear regression is Y = a + bX, where X is the explanatory variable and Y is the dependent variable. The equation selection is based on the highest correlation coefficient (*R*^2^).

## Results

### Average Involution Period of Different Parities in Holstein Cows

For the postpartum uterine involution pattern, a total of 109 Holstein dairy cows were divided into three groups based on parity. No significant difference was found in restoring the cervix uteri and non-gravid uterine horn to normal regular size after calving in biparous, primiparous, and multiparous cows. The gravid uterine horn involution period (days) of multiparous cows was 27.1 days, which was significantly shorter than that of biparous group (*P* < 0.05) (Table 1). Overall, the uterine involution of Chinese Holstein dairy cows was completed on the day 30th postpartum.

**Table 1:** Average involution period (Mean ± SEM) of the cervix, and gravid horns in different parities of Holstein cows.

**Parity**	**No. of cows**	**Cervix uteri (Days)**	**Gravid uterine horn (Days)**	**Non-gravid uterine (Days)**
Primiparous	28	30.8 ± 0.4	29.5 ± 0.4^ab^	28.9 ± 0.4
Biparous	20	30.3 ± 0.3	30.0 ± 0.6^a^	29.5 ± 0.2
Multiparous	61	29.1 ± 0.3	27.1 ± 0.4^b^	26.2 ± 0.4

*Different lowercase superscripts within a column differ significantly (P < 0.05)*.

### Average Involution Period in Holstein Cows of Different Ages

The effect of age on average involution period was identified (Table 2). The results showed that the cervical involution period was significantly longer in 2-year cows than that of older Cows. The involution period for the gravid uterine horn was gradually decreased with the increasing age. The non-gravid uterine horn's involution period for 4 and 5-year cows was significantly shorter than that of 2–3 year cows (*P* < 0.05). These results indicated that the uterine involution period was decreased with the increase of age in postpartum Holstein cows.

**Table 2:** Average involution period (Mean ± SEM) of cervix uteri and horns in a different age of Holstein cows.

**Age/years**	**No. of cows**	**Cervix uteri (Days)**	**Gravid uterine horn (Days)**	**Non-gravid uterine (Days)**
2	19	31.1 ± 0.4^a^	29.4 ± 0.4^ab^	28.9 ± 0.4^a^
3	26	30.5 ± 0.4^ab^	29.8 ± 0.4^a^	29.0 ± 0.4^a^
4	14	29.0 ± 0.4^b^	26.8 ± 0.4^c^	26.2 ± 0.8^b^
≥5	50	29.2 ± 0.3^b^	27.2 ± 0.5^bc^	26.3 ± 0.4^b^

*Different lowercase superscripts within a column differ significantly (P < 0.05)*.

### Postpartum Reproductive Tract Parameter of Holstein Cows

The influences of parity on the recovered reproductive size were identified (Table 3). The results showed that multiple calving significantly increased the cervix and non-gravid uterine diameters (*P* < 0.01; *P* < 0.05), respectively. Primiparous cows represent a relatively smaller size of the cervix uteri and non-gravid uterine horn than biparous and multiparous cows. Although there was no significant difference in gravid uterine horn between different parities (*P* > 0.05), average gravid uterine horn diameter in primiparous cows was about 0.6–1.1 mm thinner than those in biparous and multiparous cows.

**Table 3:** Average resumed diameters (Mean ± SEM) of Cervix uteri and horns in different parities of Holstein cows.

**Parity**	**No. of cows**	**Cervix uteri (mm)**	**Gravid uterine horn (mm)**	**Non-gravid uterine (mm)**
Primiparous	28	31.2 ± 0.3^*b*^	30.4 ± 0.4	29.4 ± 0.4^*b*^
Biparous	20	33.3 ± 0.3^*a*^	31.0 ± 0.3	31.0 ± 0.3^*a*^
Multiparous	61	34.1 ± 0.2^*a*^	31.5 ± 0.2	31.0 ± 0.2^*a*^

*Different lowercase superscripts within a column differ significantly, Cervix Uteri, P < 0.01; No-gravid uterine (P < 0.05)*.

Although cows over 4 years recovered faster than 2–3 year cows (Table 2), it was observed that cows over 4 years had relatively larger cervix uteri (*P* < 0.01) and non-gravid uterine horns diameters (*P* < 0.01) (Table 4). However, the resumed gravid uterine horn diameter was similar despite the different age groups. These observations were consistent with the parity effects on the resumption of reproductive tract diameters.

**Table 4:** Average involution diameter (Mean ± SEM) of uterine cervix and horns in a different age of Holstein cows.

**Age**	**No. of cows**	**Cervix uteri (mm)**	**Gravid uterine horn (mm)**	**Non-gravid uterine (mm)**
2	19	31.3 ± 0.3^b^	30.7 ± 0.3	29.3 ± 0.3^b^
3	26	31.3 ± 0.2^b^	30.2 ± 0.4	29.8 ± 0.4^ab^
4	14	34.1 ± 0.2^a^	31.5 ± 0.4	31.0 ± 0.3^a^
≥5	50	34.1 ± 0.2^a^	31.4 ± 0.2	31.1 ± 0.2^a^

*Different lowercase superscripts within a column differ significantly (P < 0.01)*.

### Postpartum Uterine Involution Pattern of the Reproductive Tract in Holstein Cows

Finally, regression curves were analyzed to show the reproductive tract's postpartum involution pattern in Holstein cows (Figures 2, 3). The regression model for the correlation between the cervix's decreased diameter and postpartum days demonstrated that the highest correlation coefficient was the quartic curve. The *R*^2^ for primiparous, biparous, and multiparous were 0.9929, 0.9733, and 0.9891, respectively. During the involution, the cervix's diameters decreased dramatically in the first 3 weeks (Day 7–21) and then declined smoothly after 3 weeks (Figure 2).

In addition, the regression model for correlation between the decrease in the diameter of the gravid uterine horn and postpartum days showed that the highest correlation coefficient was quartic curve (*R*^2^primiparous = 0.9997; *R*^2^biparous = 0.9995; *R*^2^multiparous = 0.9921) (Figure 3). The decrease in gravid horn diameters for biparous and multiparous cows was faster than for primiparous cows during the period of involution. The results showed a rapid decline in the gravid uterine horn diameter for the biparous and multiparous cows till day 16th of postpartum. However, this decline slowed down after day 16. For the primiparous cows, a decrease in the gravid uterine horn diameter reduced gradually through Holstein's involution period (Figure 3).

### Postconceptional Uterine Horn Developing Pattern From Day 30th to Day 90th

**Figure 4:**
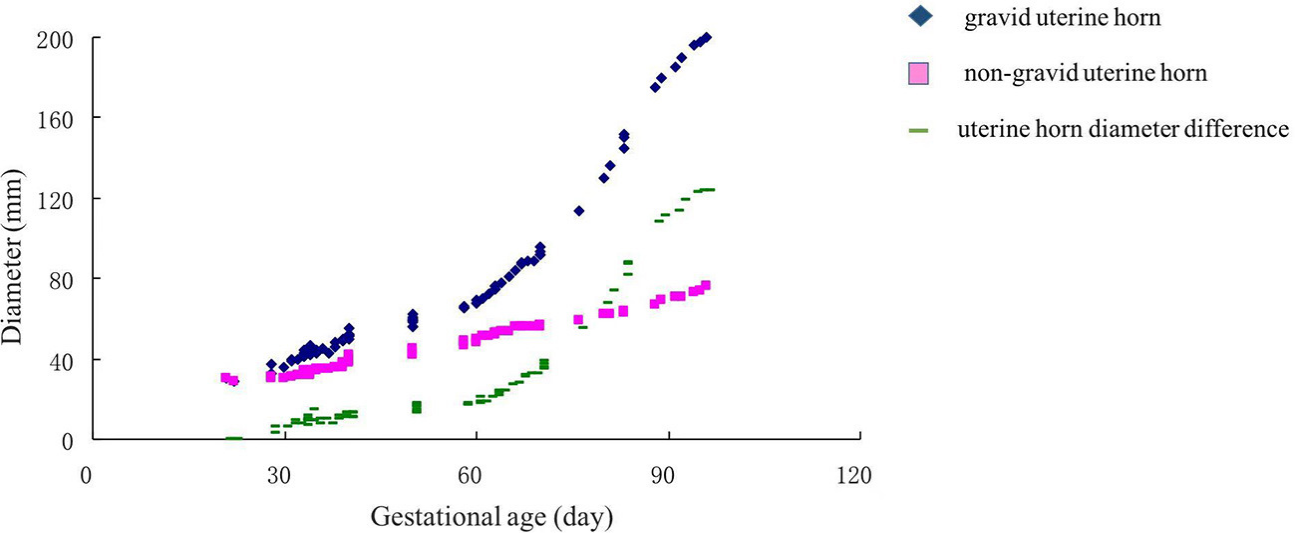
Changes in uterine horn diameters (non-gravid horn and gravid horns) in the gestation of Holstein cows. The pink square indicated a non-gravid horn diameter curve. The blue diamond indicated the gravid uterine horn. The green line showed the uterine horn diameter difference between gravid and non-gravid horns.

In the tested population, the embryonic death between 30 and 60 was 25.0%, while it was 27.3% between 60 and 90 days. The findings showed that the diameters of gravid and non-gravid uterine horns between primiparous, biparous, and multiparous cows did not vary significantly. The gravid uterine horn diameter grew slowly before 60 days, after which its diameter was dramatically increased (Figure 4). Interestingly, the diameter of the non-gravid horn was also increasing smoothly during days 30–90. The postconceptional difference between gravid and non-gravid uterine horn diameter was 32 ± 4.2 mm on day 60th and 118 ± 6.6 mm on day 90th. After using regression analysis, the formula used to determine the diameters of the gravid uterine horn in Holstein cows postconceptional on day 30th–day 90th was as follows: Gravid uterine horn diameter (mm) = −35.7564 + 2.0700 × gestational age (d) (*r* = 0.8714, *P* < 0.01).

### Postconceptional Embryonic Crown-Rump Length Growing Pattern During Day 30th to Day 90th

**Figure 5:**
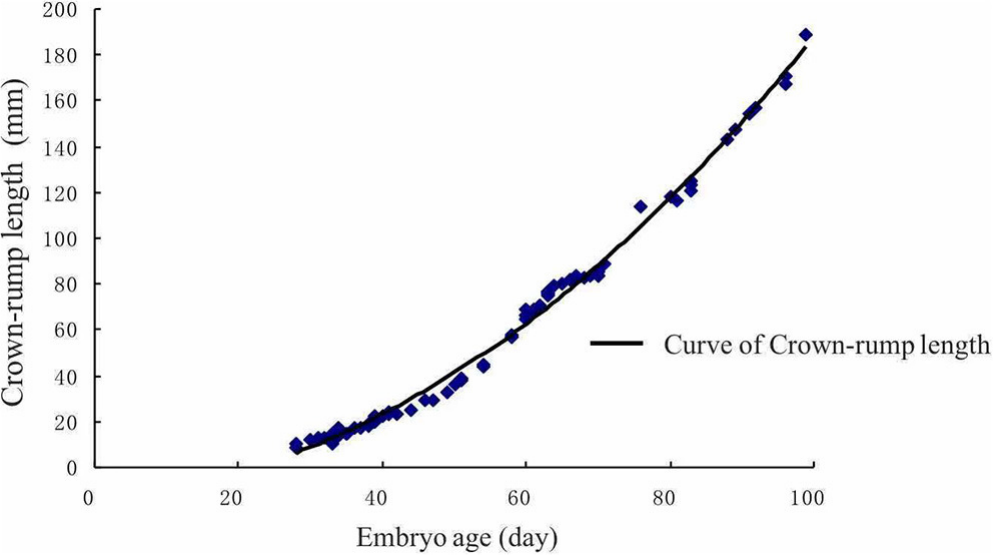
Crown-rump length of the embryo growth curve. The crown-rump size dramatically increased after the 60th day.

In this study, the crown-rump length (CRL) of the embryo dramatically increased after the 60th day (Figure 5). There was a significant positive correlation between CRL and gestational age (*r* = 0.98151, *P* < 0.01). After using regression analysis, it was shown that the formula of the CRL of embryo/fetus from 30 to 90 days was as follows: CRL (mm) = 67.19659 + 2.29815 × gestational age (d) (*r* = 0.9664, *P* < 0.01).

## Discussion

### Uterine Involution

Uterine involution period is a secondary indicator of postpartum reproductive performance. It is influenced by the negative energy balance, abnormal calving (dystocia, RFM) (32, 33). Diagnosing postpartum uterine involution is considered helpful in reducing the calving interval (4). Hence, rapid involution of the uterus could increase the overall milk yield. There are few reports of uterine involution in Chinese Holstein cows, with inconsistent effects of parity and age on uterine involution. Normally, the breed of cow influences the period of uterine involution. However, parity and age effect on uterine involution still vary within the breeds, which may be due to experimental conditions and other factors. Nutrition plays a pivotal role in determining postpartum uterine health, and poor nutritional management may lead to metabolic disorders, which delay the uterine involution (8). In addition, the uterine involution took place earlier (*P* < 0.05) in cows with BCS ≥ 3.5 than those with BCS <3.5 at birth (22). Herein, we diminished the influence of parity and age by enrolling cows with uniform BCS (3.0–3.5) and under similar nutritional management.

Rapid uterine involution in the postpartum period of dairy cows is essential to achieve early conception. Scully et al. (34), reported that it took about 49 days to complete the uterine involution of Holstein-Friesian cows in primiparous cows (34). The findings of this study, however, showed that the uterine involution was completed 29–34 days after calving, similar to 25–35 d of the involution period as reported by Hussain et al. (35). Estrus reproductive management, such as synchronization about 35 days after calving, is therefore physiologically reasonable if supplemented with an optimal nutrition plan and could shorten the calving interval of Chinese Holstein dairy cows.

Balarezo et al. reported that the time required for uterine involution in primiparous Holstein cows was significantly shorter than multiparous cows in Ecuador at a maximum temperature of 15°C (9). Our result showed that the intervals from calving to the gravid uterine horn involution in multiparous cows were shorter than those in primiparous and biparous cows in southern China with a maximum temperature of 30°C. Zhang et al. (2) findings on the uterine involution pattern of Chinese Holstein cows in northern China also support our results. These conflicts may represent the differences in climate and management systems among different studies. On the other hand, the interval from calving to the involution of the cervix uteri and the non-gravid uterine horn has no correlation between parities, consistent with the previous conclusion (2). Nevertheless, there was a considerable difference in the involution period of the cervix uteri and non-gravid horn at different age groups, which could be due to the difference of age at first mating.

Zain et al. (36) reported that the uterine involution was affected by body condition, parity, and calving season; however, calving age does not affect uterus involution. However, our research showed that the uterine involution period decreased with increasing age (Table 2). This outcome is somehow in line with many other studies; Fonseca et al. (37) reported that calving age significantly affects the uterine involution rate in Holstein and Jersey Cows. It takes an average of 33d to involute Brahman cows' uterus suggesting that similar to dairy cows calving month and age affect the involution period within beef cows (38). In the current study, the reproductive tract involution period decreased with the increase of age. While Buck et al. (39) found that the calving percentage increased from 69% of a 2.5-year beef cow to a maximum of 82% of 6–7 year beef cow and then began to decline. The difference in uterine involution caused by age may be due to malnutrition, lactation, and growth pressures in young cows, which may prolong the involution period, but not in elder cows. These results suggested that age is a critical factor that would impact the reproductive management outcome in postpartum Holstein cows.

Uterine involution, as measured by changes in each uterine horn's diameter, occurs on a decreasing logarithmic scale, which can be accurately monitored by trans rectal ultrasonography (40). The primiparous cows had the smallest diameter measurements for the non-gravid horn (*P* < 0.05) and the cervix uteri (*P* < 0.01). The results showed a rapid decline in the gravid uterine horn diameter until day 16th of postpartum for the biparous and multiparous cows. Interestingly, no significant difference was found between parity and gravid uterine horn diameter. Moreover, there was a stable positive correlation coefficient between the involution diameter of the cervix uteri and the gravid uterine horn with postnatal days in the quartic curve. However, Canadas et al. (41) argued that primiparous *(n* = 19) cows had smaller mean diameter measurements for the gravid uterine horn (*P* < 0.001) compared with multiparous (*n* = 13) cows among 34 Holstein cows when treated with eCG. Whereas, our study included primiparous (*n* = 28) and multiparous (*n* = 61) Holstein cows and had no eCG treatment. The large sample size excluding eCG treatment could be the reason for the difference in results of two studies.

In determining the interaction of age and parity on uterine involution, small sample size following stratification with parity and age was the limitation. Further studies are warranted with a large sample size to uncover the combined effect of age and parity on uterine involution.

### Embryo Development Pattern

With better management and nutrition, the milk yield of dairy cows increases steadily. However, the reproductive disorder is still one of the main problems of high yielding dairy cows (42). Besides, artificially inseminated cows have a more embryonic loss, and heat stress also negatively affects early embryonic development (43). By employing an ultrasonic scanner, it is easy to monitor the different stages of embryonic growth and development, especially in the early stages of pregnancy, by acquiring valuable information from inside the uterus (44). However, studies on Chinese Holstein cows have rarely been reported. For pregnancy detection in cows, the embryonic vesicle's sonographic images are unreliable before 20 days post-AI (44). Hence, it's recommended to start the test around 30 days post-AI (44, 45).

Using B-ultrasound, cow's early embryonic development can be observed with some distinct physiological characteristics of early embryos. According to Ali and Fahmy (46), embryo and amniotic vesicles can be observed around the fourth and fifth week of pregnancy, respectively. In addition, the organization is visible in the 7th week, while ossification is observable between the eighth and 10th week (46). Besides, Kolour et al. (44) reported that allantois, proper embryo, and heartbeat in primiparous cows are observed earlier than in multiparous cows. In the current study, uterine horn diameter clearly showed that gravid uterine horn diameters increased slowly before 60 days and increased dramatically after 60 days. Meanwhile, the diameter difference between the gravid and non-gravid uterine horn increased rapidly from 60 to 90 days. These are understandable because the organs' differentiation happened at an early stage (before 60 days), which could benefit the fetal ossification and development after the eighth week.

Gestational age is one of the most important pregnancy features and helps to estimate parturition time (47). The regression curve for Crown-rump length (CRL) may serve as an approximate standard for the normality of Holstein-Friesian fetal growth (48). It appears to be an accurate estimator of gestational age. Somnuk et al. (18) have revealed that the selected equation of CRL was: CRL (mm) = 22.679 + 12.005 gestational age (d)-1.042 gestational age (d)^2^, and the relationship between gestational age and CRL showed a strong positive correlation coefficient (*R*^2^ = 0.950) in quadratic regression. In addition, several studies have reported a strong correlation between other fetal parameters and gestational age. For example, the highest correlation was found with CRL and amniotic vesicle diameter at the early- gestation, the biparietal diameter at the mid-gestation, and the eyeball diameter at the mid-and late-gestation (46). Moreover, in a previous ultrasonic study on zebu fetuses, gestational age was highly correlated with age, weight, and length of the head, the head's circumference, front edge length of the side tail, nose-rump length, and tarsal-metatarsal length (49). However, the relationship between uterine horn diameter and gestational age is still in infancy. The current study results showed that the CRL of the embryo showed dramatic growth after day 60th, which is similar to the gravid uterine horn diameter pattern. Furthermore, we established two equations between gravid uterine horn diameters (*r* = 0.8714, *P* < 0.01), CRL (*r* = 0.9664, *P* < 0.01) and gestational age. This information could be useful for the implementation of ultrasonography in rural environments where there is no information regarding the dates of calving, estrus, or natural mating.

## Conclusions

The present study demonstrated that parity and age had significant effects on uterine involution in Chinese Holstein cows. About 30 days after calving, the Holstein cows completed the involution of the uterus. Therefore, inducing estrus around 30 days after calving under proper management can shorten the calving interval. Besides, our study established the model to estimate gestational age by uterine horn diameter and CRL with a strong positive correlation coefficient. Using both methods can reduce errors. It is essential to study the pattern of embryonic development, which is significant in predicting parturition date and making nutrition management of dairy cows.

## Data Availability Statement

The raw data supporting the conclusions of this article will be made available by the authors, without undue reservation.

## Ethics Statement

The animal study was reviewed and approved by The Ethical Committee of the Hubei Research Center of Experimental Animals [Approval ID: SCXK (Hubei) 20080005]. Written informed consent for participation was not obtained from the owners because No Written consent was required as this was a retrospective epidemiological study.

## Author Contributions

YL and HY: conceptualization. YL, HY, AA, SZ, LY, and GH: methodology. HY: software. YL, HY, and YY: investigation. YY, WY, YY, and AA: data curation. YL, MA, and GH: writing-original draft preparation. GH and HR: writing-review and editing. GH: supervision and funding acquisition. LY and GH: project administration. All authors have read and agreed to the published version of the manuscript.

## Conflict of Interest

The authors declare that the research was conducted in the absence of any commercial or financial relationships that could be construed as a potential conflict of interest.
